# Canadian wildfires are losing their climate-cooling influence from postfire snow albedo

**DOI:** 10.1073/pnas.2600434123

**Published:** 2026-06-01

**Authors:** Max J. van Gerrevink, Alemu Gonsamo, Brendan M. Rogers, Stefano Potter, Zilong Zhong, Sander Veraverbeke

**Affiliations:** ^a^ Earth and Climate, Faculty of Science, Vrije Universiteit Amsterdam, Amsterdam 1081HV, the Netherlands; ^b^ Meteorology and Air Quality Group, Wageningen University and Research, Wageningen 6700HB, the Netherlands; ^c^ School of Earth, Environment and Society, McMaster University, Hamilton, ON L8S 4L8, Canada; ^d^ Woodwell Climate Research Center, Falmouth, MA 02540; ^e^ School of Environmental Sciences, University of East Anglia, Norwich NR4 7TJ, United Kingdom

**Keywords:** boreal wildfires, biophysical impacts, albedo feedback, radiative forcing, albedo offset potential

## Abstract

The 2023 Canadian fire season was record-breaking in terms of burned area and carbon emissions. Here, we present estimates of the regional climate-cooling effect from postfire surface albedo changes, which have historically partially offset the warming influence of fire emissions by wildfires. We estimate that the 2023 fires generated a time-integrated climate cooling of –3.41 W m^−2^ of burned area (95% CI: −4.39 to −2.43) over a 70-y period. We show that the climate-cooling impact has weakened on average by 29% since the 1960s due to changes in snow cover and duration. Collectively, this result implies that modern-day boreal fires are on average twice as likely to result in a net climate-warming influence.

Canada recently experienced the largest fire season on record in terms of burned area in 2023 (1, 2), and the second largest in 2025. A top–down assessment estimated that the carbon emissions from the 2023 fire season were 647 Tg C (95% CI: 570 to 727) (2), an amount comparable to the fossil fuel emissions of high-emitting countries. In boreal forests, biogeophysical changes resulting from postfire shifts in vegetation composition, structure, and surface roughness can alter surface albedo for decades after fires. With the removal of dense, low-albedo boreal vegetation, the exposed ground, when snow-covered, reflects significantly more incoming solar radiation, especially during spring months, thereby producing a regional climate-cooling impact (3). Surface albedo tends to peak around 15 y after a fire, as regenerating vegetation remains relatively small and the burned trees have largely decomposed or collapsed, maximizing the snow exposure in spring (4, 5). Previous work has shown that, in some burned areas, the magnitude of this cooling can partially or even fully offset the warming caused by fire emissions when normalized to time-integrated radiative forcing (6–8). However, with the rapid pace of warming in high latitudes (9), fire seasons are projected to become more severe and longer (10) in tandem with declining snow cover (11, 12), which in turn may weaken the climate-cooling potential from changes in surface albedo and limit the ability to offset the radiative forcing caused by fire emissions. Here, we modeled the time-integrated climate radiative forcing resulting from changes in surface albedo associated with the 2023 fire season in Canada. Additionally, we conducted a sensitivity to climate analysis to assess how transient climate-warming since the 1960s has impacted the climate-cooling influence through changes in surface albedo to offset the fire emissions from boreal wildfires. Fire emissions here include a suite of greenhouse gases and aerosols released into the atmosphere during the combustion of aboveground biomass and organic soils. In this study, we defined the climate impacts of postfire surface albedo as the changes in net radiative flux at the top-of-atmosphere relative to a counterfactual no-fire situation, normalized by the area burned (6, 8).

## Results

**Fig. 1.**
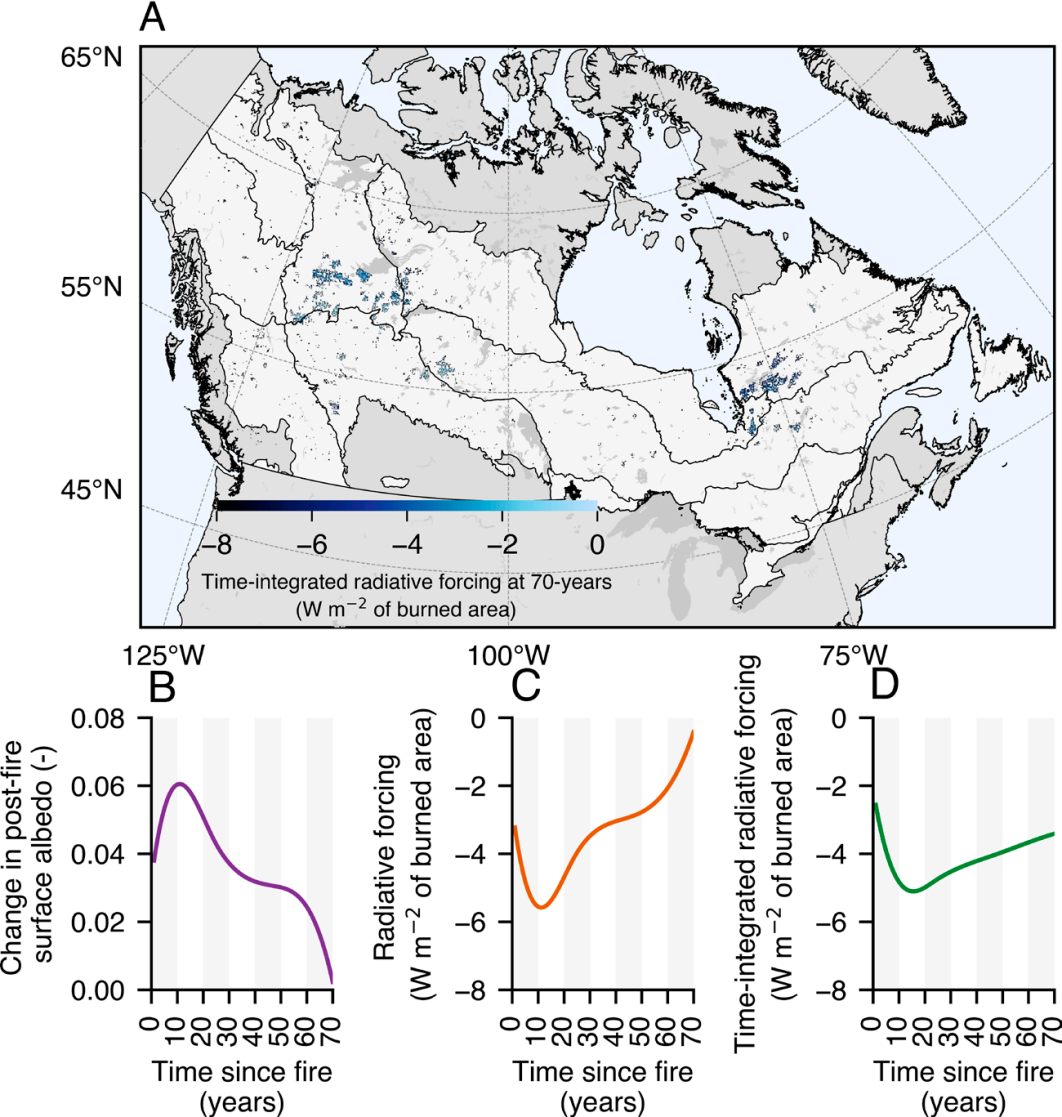
Time-integrated climate radiative forcing from changes in postfire surface albedo from the 2023 fires in Canada. (*A*) Map of 70-y time-integrated climate radiative forcing (W m^−2^ of burned area) from postfire changes in surface albedo. Trajectories of postfire surface albedo changes (*B*), median annual climate radiative forcing (*C*), and time-integrated climate radiative forcing (*D*) over the 70-y period. All trajectories are shown as splines (solid lines), fitted using cubic splines with four knots to capture nonlinear trends.

**Fig. 2.**
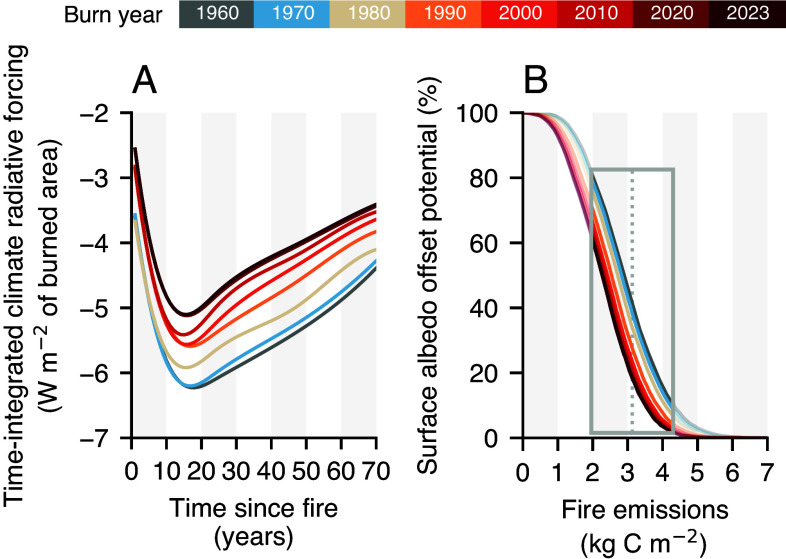
Climate sensitivity of the time-integrated climate radiative forcing from changes in postfire surface albedo. Panel (*A*) presents results from a climate sensitivity assessment, where the same burned area from 2023 was hypothetically burned in different years since 1960, illustrating how postfire albedo radiative forcing has diminished over time due to transient climate conditions, expressed in units of W m^−2^ of burned area. Panel (*B*) shows sigmoid probability curves of surface albedo offset potential indicating the probability of surface albedo to fully offset the climate-warming generated by various fire emissions. The silver box represents the average combustion rate of 3.13 ± 1.17 kg C m^−2^, whereas the dotted line represents the probability of the mean and the box boundaries the probability of one SD. The color gradient corresponds to the different burn years from 1960 through 2023.

We first constructed temporal trajectories of postfire surface albedo using monthly mean values of short-wave blue-sky albedo from the Moderate Resolution Imaging Spectroradiometer (MODIS) product (13). Machine learning models were trained using climate and environmental predictors (*SI Appendix*) to estimate postfire surface albedo at a monthly resolution over a 70-y period. Postfire albedo differences were assumed to converge to zero at year 70 (5, 6), reflecting full ecological recovery to prefire conditions. Predicted changes in surface albedo (Fig. 1*B*) were translated into annual and time-integrated climate radiative forcing estimates (Fig. 1 *C* and *D*) using monthly specific albedo radiative forcing kernels (14). We found that the strongest postfire changes in surface albedo were concentrated temporally during the second decade following the fire, whereas spatially in the coldest and northernmost regions (Fig. 1 *A* and *B*). Our estimates indicated a median time-integrated climate-cooling influence over 70-y of –3.41 W m^−2^ of burned area (95% CI: −4.39 to −2.43). However, the postfire climate-cooling impacts varied substantially across bioclimatic regions. Fires closest to the northern treeline exhibited the strongest climate-cooling effects, while approximately one-fifth of the burned area, mostly concentrated in the southern boreal forests, exhibited weaker climate cooling, with values above –2.0 W m^−2^ of burned area. These bioclimatic differences are largely determined by the snowmelt timing. Yet, when accounting for climate warming since 1960, our climate sensitivity experiment indicated that the time-integrated climate cooling has declined on average with 29% (Fig. 2*A*). This is equivalent to a reduction of 0.98 W m^−2^ of burned area at 70-y. Consequently, our results from a Monte Carlo framework suggest that the climate-cooling offset potential from surface albedo has diminished substantially, resulting in an approximate twofold reduction since 1960 (Fig. 2*B*). Contemporary boreal wildfires in North America are estimated to combust approximately 3.13 ± 1.17 kg C m^−2^ per unit area burned (15). This is a conservative estimate as it does not account for postfire permafrost thaw emissions. We found that the surface albedo offset potential was substantially higher under historical climate conditions (Fig. 2*B*). The albedo offset potential declined from 41.2% (95% CI: 11.2 to 80.0%) in 1960 to 18.2% (95% CI: 1.9 to 59.8%) in 2023 based on average combustion rates (Fig. 2*B*). These results show that whereas changes in surface albedo once fully compensated warming across approximately two-fifths of the burned area, this is predicted to be the case for approximately only one-fifth of the burned area in 2023 under shared socioeconomic pathway (SSP) 2-4.5.

## Discussion

In this Article, we estimated the time-integrated radiative forcing over a 70-y period for the 2023 fire season in Canada under SSP2-4.5. We found that postfire changes in surface albedo contribute to a moderate but regionally substantial climate-cooling influence on the climate of –3.41 W m^−2^ of burned area (95% CI: −4.39 to −2.43), comparable to previous findings from field-based surface albedo measurements (6, 7). Long-lasting spring snow exposure is sustaining this climate-cooling influence. Randerson et al. (6) documented an average climate cooling of –4.2 ± 2.0 W m^−2^ of burned area over 80-y in Interior Alaska, while O’Halloran et al. (7) found a similar climate-cooling influence over a 70-y period in central Canada. Unlike field-based studies that rely on limited in situ measurements, our remote sensing approach captures broader spatial heterogeneity and temporal dynamics. This may help explain the broader observed range and spatial patterns, as it reflects both methodological differences and localized environmental conditions. Moreover, our estimates fall within the spatial ranges reported by previous remote sensing studies, particularly when normalized by the area burned (4, 5). Nonetheless, the spatially heterogeneous patterns highlight that postfire surface albedo climate feedbacks are not uniform and are strongly controlled by regional differences in snow cover, vegetation, soil properties, and fire severity.

Historically, the climate-cooling influence from increased surface albedo driven by prolonged snow exposure has played a dominant role in the net climate impacts from boreal fires (6, 7). Here, we demonstrate that the magnitude of albedo-induced cooling is sensitive to ongoing climate change and has declined with 29% since 1960 (Fig. 2*A*). This trend reflects the earlier snow disappearance rates, later snow onset, and warming temperatures in the Arctic-boreal zone (11, 12). Importantly, alterations in postfire surface albedo constitute just one component of the climate radiative forcing balance from boreal wildfires. Higher postfire albedo cooling does not necessarily correspond to greater carbon combustion at a continental scale, where fires near the northern treeline exhibit the strongest postfire snow albedo signal, yet also have comparatively lower carbon consumption per unit area. At a more regional scale, however, remotely sensed proxies of carbon combustion have correlated reasonably strongly with postfire albedo changes (4, 8). With continued climate change, warmer and drier weather conditions will increase the vulnerability of Canadian boreal ecosystems to severe and longer fire seasons (16), which in turn can burn deeper into organic soil layers, thereby potentially consuming parts of the legacy carbon pool (17), while the cooling from surface albedo will continue to decline. This indicates that the probability for net climate-cooling impacts of wildfires has progressively become smaller, and that fires with an average combustion of 3.13 kg C m^−2^ of burned area are approximately twice as likely to result in net climate-warming compared to 1960. For the southern boreal fires, earlier snow disappearance rates will reduce the surface albedo offset potential faster. The transition highlights the growing role of boreal fires as a catalyzer in a positive climate feedback loop, with net climate-warming fires progressively migrating northward. Together, our findings reveal a critical shift, as boreal fires are transitioning from a historically climate-cooling biogeophysical influence into a net warming influence from fire emissions and a reduction in their biogeophysical cooling impacts from surface albedo.

## Materials and Methods

To map the 2023 fire season in Canada, we used the Landsat-based 30 m burned area product by Pelletier et al. (1), spatially averaged to a 500 m grid to normalize climate forcing estimates per unit of burned area. Time-integrated climate radiative forcing was estimated over a 70 y postfire period under climate change scenario SSP2-4.5. Model estimates were converted into radiative forcing using monthly surface albedo radiative forcing kernels (14), and expressed in W m^−2^ of burned area. Full methods are provided in *SI Appendix*.

## Supplementary Material

Appendix 01 (PDF)

## Data Availability

Codes data have been deposited in public access is granted below: https://zenodo.org/records/19221976 (18). Previously published data were used for this work [https://data.nasa.gov/dataset/above-modis-derived-daily-mean-blue-sky-albedo-for-northern-north-america-2000-2017-7fc49/resource/c839b68d-c2ff-4ac2-be2f-4997b64b7709 (13)]. All other data are included in the manuscript and/or *SI Appendix*.
