# Supplementary material for: Canadian wildfires are losing their climate-cooling influence from postfire snow albedo
Source: Proc Natl Acad Sci U S A. 2026 Jun 1;123(23):e2600434123. doi: 10.1073/pnas.2600434123 (PMC13250525; doi:10.1073/pnas.2600434123)
Supplement: Supplementary file 1 — Appendix 01 (PDF) [file pnas.2600434123.sapp.pdf]

Supporting Information for

Canadian wildfires are losing their climate-cooling influence from post-fire snow albedo

Max J. van Gerrevink, Alemu Gonsamo, Brendan M. Rogers, Stefano Potter, Zilong Zhong, and Sander Veraverbeke

Max J. van Gerrevink
Email: m.j.van.gerrevink@vu.nl

This PDF file includes:

Extended methods
SI References

Extended methods

To assess the long-term biogeophysical climate radiative forcing from surface albedo changes following the 2023 Canadian fire season, we adopted the framework from Potter et al. (1). This framework integrates satellite-derived surface albedo data, climate and environmental datasets to predict post-fire surface albedo trajectories across boreal North America and the associate climate forcing. In the main text and extended methods, we report time-integrated radiative forcing as the influence of burned area on the net radiative flux at the top-of-atmosphere (2–5), normalized by the burn fraction within a 500 m pixel. We report time-integrated spatially explicit climate radiative forcing estimates under historical and ongoing transient climate change using shared socioeconomic pathway SSP2-4.5 (6).

Temporal modeling of post-fire albedo trajectories

To understand the long-term post-fire surface albedo dynamics for boreal North America, we used a retrospective space-for-time approach (7). We combined the fire databases from Alaska (8) and Canada (9, 10) to create a comprehensive boreal North American fire record that extends beyond the temporal coverage of remote sensing products. By linking historical fire perimeters to remote sensing derived surface albedo observations, we assembled a spatial and temporal mosaic of observations representing various stages of post-fire surface albedo up to a 70-year post-fire environment. We included observations from Alaska to enhance the robustness and scalability of our approach, expanding the ecological and climatic gradient represented in the post-fire surface albedo trajectories. The 70-year post-fire period approximates the full recovery timescale of surface albedo across boreal forest ecosystems (1, 11). However, this is primarily constrained by the availability and reliability of historical fire records, as in Alaska, records extend back to the 1920s but contain omissions in earlier decades, as for in Canada, systematic fire reporting began in the early 1960s (8–10).

We used monthly aggregates of the MODIS-derived daily mean short wave blue-sky albedo product between 2000-2017 (12). This product was derived from the MODIS MCD43A1 V006 Bidirectional Reflectance Distribution Function and Albedo (BRDF/Albedo) product (13). We retained surface albedo observations with full inversions flagged as best or good quality, along with valid aerosol optical depth (AOD) measurements derived from either the MOD08 V6 Atmosphere Daily and Monthly Global Product Bands or the MISR Level 3 Component Monthly Global Aerosol Product (MIL3MAE) for the months May through September. For the remaining months, we limited our selection to best or good quality MCD43A1 pixels without applying any AOD constraints. The one exception was December, which did not produce reliable observations due to high solar zenith angles. We therefore estimated December albedo by averaging the monthly means from January and November.

The retrospective space-for-time dataset included 47,554 fire perimeters in total, comprising 43,791 fires from the Canadian National Fire Database and 3,763 fires from the Alaska Large Fire Database, spanning 1940 to 2016. Nearly 30% of all fire perimeters (14,091 fires in total) predates 1980, providing sufficient amount of observations necessary to constrain the long-term recovery trajectory of surface albedo. The majority of fire perimeters before 1980 (13,348 fires) were located in Canada. The number of valid pixels varies per post-fire year and monthly model due to our blue-sky albedo selection criteria. The number of included pixels ranged from 2,292 to 31,149 pixels in January to 127,134 to 1,859,811 pixels in June. The difference reflects the higher frequency of clear-sky observations during summer months. Simultaneously, the sampling density decreases with time since fire, reflecting the declining availability of older fire perimeters in the historical records.

Using our space-for-time dataset, we built separate random forest regressor models (14) to predict the post-fire surface albedo for each post-fire month and year using 32 bioclimatic and 15 environmental datasets. We included high spatial resolution bioclimatic data from ClimateNA. ClimateNA uses high-resolution Parameter-elevation Regressions on Independent Slopes Model and WorldClim Global Climate data to represent historical climate normals across North America at a spatial resolution of 1km (15). For the training data, we used historical long-term climate

normals representative of the 1981-2010 epoch (15). We used long-term climate normals over interannual time series, as the latter would mix year-to-year climatic variability with the post-fire successional signal that the model is intended to represent. In addition, we used environmental features related to topography (16), permafrost (17), and soil characteristics (18). The models were trained on all features using an 80/20 train-test split. We then applied feature selection to retain the ten most influential predictors. The final model predicted post-fire surface albedo using permafrost zonation index, ruggedness index, elevation, soil pH, silt content, climate moisture deficit, evaporation, and autumn and spring temperature and precipitation (15–18). The hydrobioclimatic variables climatic moisture deficit and evaporation, are included as standard outputs within the ClimateNA dataset and were derived using Hargreaves' reference evaporation method (19). All datasets were resampled using nearest neighbor interpolation techniques to match the spatial resolution of 500 m.

Machine learning models were trained to predict post-fire surface albedo at monthly resolution over a 70-year period. Model performance was lowest in January and February, with median R^2 values of 0.75 (± 0.046) and 0.76 (± 0.051). Accuracy improved during the spring and summer months, reaching a median R^2 of 0.88 (± 0.03) in April, and remained high through the growing season. Performance declined slightly in early autumn, with R^2 values of 0.84 (± 0.028) in September and 0.82 (± 0.028) in October, before peaking in November at 0.90 (± 0.05). Across all months, model consistency was high, with monthly standard deviations ranging from 0.026 to 0.051. Root mean square error values followed a similar seasonal pattern, with the lowest errors in summer (95% CI = 0.0077-0.0091) and higher uncertainty in winter (95% CI = 0.036-0.059).

To account for future climate change in our predictions, we substituted the historical bioclimatic datasets used in training, with climate projections from the SSP2-4.5 climate scenario. We obtained the climate projections from ClimateNA using the ensemble mean of 13 Coupled Model Intercomparison Project Phase 6 atmosphere-ocean general circulation models (15). This data is provided in four distinctive epochs of 20-years, for which we implemented a stepwise epoch-based substitution. Each post-fire year was assigned climate variables from the epoch it fell within, ensuring that modeled post-fire surface albedo reflect the transient climate conditions projected under SSP2-4.5. The environmental datasets such as elevation (16), permafrost zonation (17), and soil parameters (18) were treated as static inputs, assuming no change in the post-fire period.

Calculating time-integrated radiative forcing in W m^{-2} of burned area

We used our post-fire predictions of surface albedo to calculate changes in comparison to a no-fire situation under evolving climate conditions. We assumed that the difference between post-fire and no-fire conditions converged to zero after 70 years, reflecting full ecological recovery. Thus, we calculated the changes in surface albedo by differencing the predicted post-fire surface albedo at each time step from the 70-year post-fire estimate. We then converted surface albedo changes into changes in radiative flux at the top-of-atmosphere (TOA) using monthly spatially explicit kernels from Pendergrass et al. (20). The kernels express the changes in TOA radiative flux in W m^{-2} %$^{-1}$ change in surface albedo at a resolution of 0.25°. We resampled the albedo kernels to match our 500 m spatial grid using nearest neighbor interpolation and aggregated the estimates in monthly flux changes to report annual climate radiative forcing for each post-fire year. From the annual estimates of albedo radiative forcing, we computed a time-integrated metric in order to capture the cumulative effects of post-fire surface albedo changes over the 70-year period (1). Our results were normalized by the burn fraction within each 500 m grid to express the climate radiative forcing per unit burned area. We mapped the climate impacts using a Landsat-based 30 m burned area product from the 2023 Canadian fire season by Pelletier et al. (21). This product was resampled to a 500 m grid and used for the post-fire surface albedo predictions and to calculate the burn fraction. We computed the burn fraction as the proportion of 30 m grid cells classified as burned within each 500 m pixel, resulting in a value between zero and one.

Sensitivity to climate assessment and estimating the albedo offset potential

We conducted a sensitivity to climate analysis to assess how transient climate warming since 1960 has impacted the climate-cooling influence from post-fire surface albedo changes. To isolate the

effect of climate change, we held the spatial extent of the 2023 Canadian fire season constant and experimentally varied the burn year to model the same fires under historical bioclimatic conditions. Specifically, fire pixels were experimentally burned in the years: 1960, 1970, 1980, 1990, 2000, 2010 and 2020. For these burn years we implemented the full time-integrated climate radiative forcing framework described above for all burned pixels in our 500 m grid. Bioclimatic variables used to predict the post-fire surface albedo were replaced with data to correspond to historical climate epochs from 1961 to 1990 and 1991 to 2020 (15). Yearly climate values were assigned to each post-fire year based on its corresponding epoch, ensuring that modeled post-fire surface albedo reflects transient climate conditions. We further used these results to understand how climate change has impacted the probability of surface albedo potential to offset the fire emissions from boreal forest fires. In this Article, we defined the total fire emissions as a suite of greenhouse gas and aerosol emissions from the combustion of aboveground biomass and organic soils. We combined the radiative impacts from well-mixed greenhouse gases (CO_2, CH_4, N_2O and O_3), precursors (NO_x, CO, non-methane volatile organic compounds (NMVOCs)), and aerosol (black and organic carbon) emissions. In doing so, we built a temporally explicit climate radiative forcing box model (22, 23). Our framework incorporated well-mixed greenhouse gases using simplified radiative expressions (4). We accounted for the variable CO_2 lifetime using concentration-based impulse-response functions (24). For CH_4, we applied a fixed atmospheric lifetime of 12.4 years, while for N_2O we accounted for the feedback of future N_2O concentrations on its own lifetime (25). Radiative forcing from ozone was derived as a function of fire-related CO emissions (26, 27). For shorter-lived climate forcers such as NO_x, CO, NMVOCs, and organic and black carbon aerosols, we adopted a time-integrated approach based on global warming potentials (GWPs) relative to CH_4 at 20- and 100-year horizons (22, 23, 28, 29). GWP-to-radiative-forcing conversion factors for organic and black carbon were derived by referencing their GWPs against that of CH_4, for which direct forcing estimates are available. Because aerosols are removed from the atmosphere within weeks via dry and wet deposition, their total forcing was assigned to the first post-fire year. Indirect aerosol forcing was estimated by scaling direct aerosol forcing by the ratio of indirect to all-sky direct radiative forcing from aerosol emissions (30). Together, these formulations allowed us to simulate the cumulative radiative forcing efficiency per kilogram of carbon combusted over a 70-year post-fire period under SSP2-4.5 conditions.

The surface albedo offset potential was defined as the proportion of fire emissions, expressed in units of kg C m^{-2}, that could be counterbalanced by the climate-cooling effect from the changes in post-fire surface albedo. This is calculated by the following equation:

$$Surface\ albedo\ offset\ potential\ (\%) = \frac{\Delta A}{\lambda_C \times \epsilon_C} \times 100\%$$

Where:
ΔA: Time-integrated climate radiative forcing, expressed in units of W m^{-2} of burned area,
λ_C : Radiative forcing efficiency of one kilogram carbon combusted, expressed in units of W m^{-2},
ϵ_C : Fire emissions, expressed in units of kg C m^{-2}.

We implemented a Monte Carlo simulation of 10,000 runs to account for the uncertainty in all three components of the surface albedo offset potential equation. Instead of calculating offset potential for a single fire emission value, we created a continuous range of combustion rates ranging from 0.01 to 7.0 kg C m^{-2}. Because of the shortage in a fire carbon combustion product for the 2023 Canadian wildfires at a spatial resolution matching our albedo assessment, we used a range of combustion values that represent the possible magnitude of fire emissions from Canadian fires (31). We calculated the proportion of each run where the surface albedo offset potential exceeded 100%. This proportion is the likelihood of full offset, meaning the probability that surface albedo-induced cooling completely compensates climate-warming from fire emissions. These probabilities were plotted against the combustion rates, producing sigmoid curves that describe the transition from high likelihood of full offset at low emissions to near-zero likelihood at high emissions. We included each burn year using the median time-integrated climate radiative forcing value at 70 years and the domain wide relative uncertainty value of 14.6% (see section on model uncertainty). Regarding the amount of carbon combustion, we included a domain-wide standard deviation

estimate of combustion derived from synthesis and modeling estimates across central and western boreal North America of 1.17 kg C m^{-2} per unit area burned (31). The radiative forcing efficiency was derived by propagating one kilogram of carbon through the temporally explicit climate radiative forcing model (22, 23, 32). When tailored to the 2023 fire season, one kilogram carbon combustion results on average in 1.56 W m^{-2} over a 70-year period, with a relative uncertainty margin of 36%. Noteworthy, this radiative forcing efficiency value does not account for post-fire carbon uptake by recovering boreal vegetation.

Model limitations and uncertainty

The surface albedo model presented in this study uses a space-for-time substitution approach, in which spatial relationships between climate variables, environmental variables and post-fire surface albedo are used to project the response under the SSP2-4.5 climate scenario. This approach assumes that the surface albedo relationships observed across space during the training period are transferable to future temporal conditions. Several key limitations are relevant here. First, the reference period of the climate data (1981-2010 long-term climate normals) is representative for a specific and bounded climate space. Future climate projections are expected to include climate conditions that fall outside the 1981-2010 training envelope. This will be most notably in regions near the treeline (33). Predictions in these regions therefore represent extrapolation beyond the calibrated model space, and thus carry more uncertainty. Second, our model treats the permafrost extent as a static variable, reflecting contemporary permafrost conditions (17). However, under future warming, permafrost may thaw substantially, changing the post-fire active layer thickness and vegetation recovery trajectories in ways that are not captured in the current model. This may result in overestimation of the surface albedo cooling impacts across permafrost-dominated landscapes. We did not explicitly account for alternative pathways of forest regeneration. Where biotic legacies are lost or abiotic conditions are misaligned with dominant species traits, delays or alternative pathways of forest regeneration can occur (34). Increased dominance of deciduous stands after fires may change the allocation of carbon in above- and belowground carbon pools (35, 36), and locally result in a non-winter climate-cooling from albedo signal of -0.038 ± 0.006 W m^{-2} (one standard deviation), when compared to a deterministic vegetation recovery trajectory (37). This represents a relative uncertainty of roughly 1.12% (95% CI: 0.66 to 1.58%) on our median surface albedo cooling estimate of -3.41 W m^{-2} of burned area (95% CI: −4.39 to −2.43). While omitting alternative recovery pathways may lead to a slight underestimation of the cooling impacts, this uncertainty is small compared to the overall signal from snow albedo. Lastly, we did not account for the carbon uptake from recovering vegetation or fire-induced carbon emissions related to permafrost thaw in our surface albedo offset potential calculations. These two components exert opposing influences on climate, as vegetation regrowth would exert a cooling influence and permafrost-related carbon emissions a warming influence. However, van Gerrevink et al. (22) show that these components are comparable in magnitude. Their findings demonstrate that carbon uptake would account for roughly 9.5% and fire-induced permafrost emissions for roughly 10.6% of the radiative impacts from combustion emissions over a 70-year post-fire period. As these factors largely counterbalance each other, their omission is unlikely to substantially alter our conclusions.

Model uncertainty was estimated using a bootstrapping approach applied to the ensemble of decision trees in each random forest regressor model. This generated 500 bootstrap realizations per pixel by resampling the tree-level predictions with replacement. We propagated each realization through the full time-integrated climate radiative forcing pipeline and subsequently calculated the relative uncertainty of model predictions as the coefficient of variation. For domain-wide uncertainty, we computed the median prediction for each bootstrap realization, resulting in 500 median values. We tested the distribution of these medians for normality using Shapiro-Wilk and Anderson-Darling tests to assess whether a parametric confidence interval would be appropriate. These tests indicated no significant deviation from normality ($p > 0.05$). Based on this, we reported domain-wide uncertainty as a parametric 95% confidence interval derived from a normal approximation, using the average coefficient of variation across all pixels. We did not account for the inclusion of unburned islands within the perimeters of the historical fire records that may introduce observations of unburned pixels in our post-fire surface albedo chronosequence. Unburned islands may be

influential for fire perimeters earlier in the record. Additionally, we did not account for uncertainty in the monthly TOA albedo radiative forcing kernels.

Code availability

All analyses were conducted using Python software 3.11.10. The code deemed central for the analyses in this paper is available via: https://zenodo.org/records/19221976.

SI References

1. S. Potter, *et al.*, Climate change decreases the cooling effect from postfire albedo in boreal North America. *Glob. Chang. Biol.* **26**, 1592–1607 (2020).
2. G. Myhre, D. Shindell, Chapter 8 : Anthropogenic and Natural Radiative Forcing.1–124 (2011).
3. G. Myhre, E. J. Highwood, K. P. Shine, F. Stordal, New estimates of radiative forcing due to well mixed greenhouse gases. *Geophys. Res. Lett.* **25**, 2715–2718 (1998).
4. M. Etminan, G. Myhre, E. J. Highwood, K. P. Shine, Radiative forcing of carbon dioxide, methane, and nitrous oxide: A significant revision of the methane radiative forcing. *Geophys. Res. Lett.* **43**, 12,614-12,623 (2016).
5. P. Forster, *et al.*, "The Earth's Energy Budget, Climate Feedbacks and Climate Sensitivity" in Climate Change 2021: The Physical Science Basis. Contribution of Working Group I to the Sixth Assessment Report of the Intergovernmental Panel on Climate Change, *Cambridge University Press.* 923–1054 (2021).
6. M. Meinshausen, *et al.*, The shared socio-economic pathway (SSP) greenhouse gas concentrations and their extensions to 2500. 3571–3605 (2020).
7. R. S. L. Lovell, S. Collins, S. H. Martin, A. L. Pigot, A. B. Phillimore, Space-for-time substitutions in climate change ecology and evolution. *Biological Reviews* **98**, 2243–2270 (2023).
8. E. S. Kasischke, D. Williams, D. Barry, Analysis of the patterns of large fires in the boreal forest region of Alaska. *Int. J. Wildland Fire* **11**, 131–144 (2002).
9. B. J. Stocks, *et al.*, Large forest fires in Canada, 1959-1997. *Journal of Geophysical Research: Atmospheres* **108** (2002).
10. B. D. Amiro, *et al.*, Direct carbon emissions from Canadian forest fires, 1959-1999. *Canadian Journal of Forest Research* **31**, 512–525 (2001).
11. J. T. Randerson, *et al.*, The impact of boreal forest fire on climate warming. *Science.* **314**, 1130–1132 (2006).
12. K. K. Solvik, *et al.*, ABoVE: MODIS-Derived Daily Mean Blue Sky Albedo for Northern North America, 2000-2017. *ORNL DAAC, Oak Ridge, Tennessee, USA.* (2019). https://doi.org/https://doi.org/10.3334/ORNLDAAC/1605.
13. Z. Schaaf, C., Wang, MCD43A1 MODIS/Terra+Aqua BRDF/Albedo Model Parameters Daily L3 Global - 500m V006 [Data set]. *NASA EOSDIS Land Processes DAAC.* (2015).
14. L. Breiman, Random Forests. *Mach. Learn.* **45**, 5–32 (2001).
15. C. R. Mahony, T. Wang, A. Hamann, A. J. Cannon, A global climate model ensemble for downscaled monthly climate normals over North America. *International Journal of Climatology* **42**, 5871–5891 (2022).
16. J. J. Danielson, D. B. Gesch, "Global multi-resolution terrain elevation data 2010 (GMTED2010)" (US Geological Survey, 2011).
17. S. Gruber, Derivation and analysis of a high-resolution estimate of global permafrost zonation. *Cryosphere* **6**, 221–233 (2012).
18. T. Hengl, *et al.*, SoilGrids250m: Global gridded soil information based on machine learning. *PLoS One* **12** (2017).
19. G. H. Hargreaves, Z. A. Samani, Reference crop evapotranspiration from temperature. *Appl. Eng. Agric.* **1**, 96–99 (1985).
20. A. G. Pendergrass, A. Conley, F. M. Vitt, Surface and top-of-Atmosphere radiative feedback kernels for cesm-cam5. *Earth Syst. Sci. Data* **10**, 317–324 (2018).
21. F. Pelletier, J. A. Cardille, M. A. Wulder, J. C. White, T. Hermosilla, Revisiting the 2023 wildfire season in Canada. *Science of Remote Sensing* **10** (2024).
22. M. J. van Gerrevink, *et al.*, Climate impacts from North American boreal forest fires. *Nat. Geosci.* (2026). https://doi.org/10.1038/s41561-026-01940-3.
23. M. Moubarak, S. Sistla, S. Potter, S. M. Natali, B. M. Rogers, Carbon emissions and radiative forcings from tundra wildfires 1 in the Yukon-Kuskokwim River Delta, Alaska 2. *Biogeosciences* (2023). https://doi.org/10.5194/bg-2022-144.

24. F. Joos, *et al.*, Carbon dioxide and climate impulse response functions for the computation of greenhouse gas metrics: A multi-model analysis. *Atmos. Chem. Phys.* **13**, 2793–2825 (2013).
25. M. Meinshausen, S. C. B. Raper, T. M. L. Wigley, Emulating coupled atmosphere-ocean and carbon cycle models with a simpler model , MAGICC6 – Part 1 : Model description and calibration. *Atmos. Chem. Phys.* 1417–1456 (2011). https://doi.org/10.5194/acp-11-1417-2011.
26. D. A. Jaffe, N. L. Wigder, Ozone production from wild fires : A critical review. *Atmos. Environ.* **51**, 1–10 (2012).
27. D. S. Stevenson, *et al.*, Tropospheric ozone changes, radiative forcing and attribution to emissions in the Atmospheric Chemistry and Climate Model Intercomparison Project (ACCMIP). *Atmos. Chem. Phys.* **13**, 3063–3085 (2013).
28. M. M. Fry, *et al.*, The influence of ozone precursor emissions from four world regions on tropospheric composition and radiative climate forcing. *Journal of Geophysical Research Atmospheres* **117** (2012).
29. W. J. Collins, *et al.*, Global and regional temperature-change potentials for near-term climate forcers. *Atmos. Chem. Phys.* **13**, 2471–2485 (2013).
30. D. S. Ward, *et al.*, The changing radiative forcing of fires: Global model estimates for past, present and future. *Atmos. Chem. Phys.* **12**, 10857–10886 (2012).
31. S. , Potter, *et al.*, Daily Burned Area and Carbon Emissions Across Western Boreal North America between 2001 and 2019. *Biogeosciences* **20**, 2785–2804 (2023).
32. M. J. van Gerrevink, *et al.*, Code files for “Climate impacts from North American boreal forest fires”. (2025). Available at: https://doi.org/10.5281/zenodo.15113299.
33. S. Potter, *et al.*, Climate change decreases the cooling effect from postfire albedo in boreal North America. *Glob. Chang. Biol.* **26**, 1592–1607 (2019).
34. J. F. Johnstone, *et al.*, Changing disturbance regimes, ecological memory, and forest resilience. *Front. Ecol. Environ.* **14**, 369–378 (2016).
35. J. L. Baltzer, *et al.*, Increasing fire and the decline of fire adapted black spruce in the boreal forest. *Proceedings of the National Academy of Sciences* **118**, 1–9 (2021).
36. M. C. Mack, *et al.*, Carbon loss from boreal forest wildfires offset by increased dominance of deciduous trees. *Science (1979).* **372**, 280–283 (2021).
37. R. Massey, *et al.*, Forest composition change and biophysical climate feedbacks across boreal North America. *Nat. Clim. Chang.* **13**, 1368–1375 (2023).
